# S100A1 expression characterizes terminally differentiated superficial cells in the urothelium of the murine bladder and ureter

**DOI:** 10.1007/s00418-022-02120-1

**Published:** 2022-06-01

**Authors:** Fairouz Qasrawi, Max Meuser, Finja Lehnhoff, Marjenna Schulte, Andreas Kispert

**Affiliations:** Institut Für Molekularbiologie, OE5250, Medizinische Hochschule Hannover, Carl-Neuberg-Str. 1, 30625 Hannover, Germany

**Keywords:** Urothelium, Bladder, Ureter, Superficial cells, S100A1, Differentiation

## Abstract

The urothelium is a stratified epithelium that lines the inner surface of the components of the urinary drainage system. It is composed of a layer of basal cells, one or several layers of intermediate cells, and a layer of large luminal superficial or umbrella cells. In the mouse, only a small set of markers is available that allows easy molecular distinction of these urothelial cell types. Here, we analyzed expression of S100A1, a member of the S100 family of calcium-binding proteins, in the urothelium of the two major organs of the murine urinary tract, the ureter and the bladder. Using RNA in situ hybridization analysis, we found exclusive expression of *S100a1* mRNA in luminal cells of the ureter from embryonic day (E)17.5 onwards and of the bladder from E15.5 to adulthood. Immunofluorescence analysis showed that expression of S100A1 protein is confined to terminally differentiated superficial cells of both the ureter and bladder where it localized to the nucleus and cytoplasm. We conclude that S100A1 is a suitable marker for mature superficial cells in the urothelial lining of the drainage system of the developing and mature mouse.

**Supplementary Information:**

The online version contains supplementary material available at 10.1007/s00418-022-02120-1.

## Introduction

The urothelium is a stratified epithelium that lines the inner surface of the components of the urinary drainage system, i.e., of the paired renal pelvises and ureters, and the unpaired bladder and the proximal urethra. Within the ureter and bladder, the urothelium is composed of three major cell types that are organized in radial layers of variable thickness. Bordering the lumen are large bi- or multinucleated terminally differentiated superficial (S-) or umbrella cells that primarily account for the barrier function of this tissue, partly by the formation of tight junctions between them, and partly by the expression of uroplakins (UPKs), that assemble as heterotetramers to form crystalline plaques on the apical surface (Hu et al. 2002; Acharya et al. 2004; Sun et al. 1996). Underneath the S-cells are much smaller intermediate (I-) cells that form layers of one to several cell thickness and serve as precursors (Gandhi et al. 2013; Bohnenpoll et al. 2017). Sitting on the basement membrane is a layer of cuboidal basal (B-) cells that are smallest in size but constitute the most abundant population of cells in the mature tissue (for reviews see Arrighi 2015; Lazzeri 2006; Dalghi et al. 2020)).

In addition to size and location, a small set of proteins have been established whose combinatorial expression distinguishes the three urothelial cell types (Dalghi et al. 2020). B-cells uniquely express the intermediate filament protein KRT5. They are also positive for expression of the ∆N isoform of the runt-type transcription factor TRP63 (∆NP63) but negative for UPKs. I-cells are KRT5 negative and express low levels of ∆NP63 and UPKs, whereas S-cells lack expression of KRT5 and ∆NP63 but express high levels of UPKs (Gandhi et al. 2013; Bohnenpoll et al. 2017). S-cells in the mature bladder also express KRT20 (Erman et al. 2001).

Expression analysis of these markers at embryonic stages revealed a staggered differentiation of the urothelial cell types in the development of the murine bladder and ureter. In the cloaca, the bladder primordium, epithelial progenitors start to stratify at embryonic day (E)12.5, concomitant with the expression of ∆NP63. Cells of the luminal layer express UPKs at E14.5, and increase their DNA content and polyploidy by incomplete cytokinesis and endoreplication, indicating S-cell differentiation (Wang et al. 2018). B-cell differentiation occurs at E16.5 as visualized by expression of KRT5 in the basal layer (Gandhi et al. 2013). This sequence of marker activation is preserved in ureter development but occurs with a delay of 2 days (Bohnenpoll et al. 2017).

S100 proteins are a vertebrate-specific family of small dimeric calcium-binding proteins. They have two EF-hands, which upon calcium binding trigger structural changes in the protein that allow interactions with a large cohort of target proteins and modulations of their activity. The physiological functions of S100 proteins are poorly understood, but intracellularly they are known to participate in processes such as proliferation, differentiation, apoptosis, Ca^2+^ homeostasis, and energy metabolism, while extracellular S100 proteins can activate a variety of receptors (for reviews see Marenholz et al. 2004; Weisz and Uversky 2020; Ecsédi et al. 2021).

Altered S100 mRNA or protein levels have been described in various pathologies such as cardiomyopathies, neurodegenerative and inflammatory disorders, and cancers (Weisz and Uversky 2020; Ecsédi et al. 2021). In mouse urothelial carcinoma, *S100a1* and *S100a5* were underexpressed, whereas *S100a3, S100a8, S100a9*, *S100a10*, and *S100g* where overexpressed (Yao et al. 2007a). Nucleocytoplasmic immunoreactivity of S100P was detected in normal human urothelium and in urothelial carcinoma cells (Higgins et al. 2007). Later work confirmed the high prevalence of S100P positivity in high-grade urothelial carcinomas (reviewed in (Suryavanshi et al. 2017).

S100A1 is the founding member of the S100 protein family. It is expressed in many tissues including the nervous system, the developing and mature heart, skeletal muscle, and kidney (Zimmer et al. 1995). As a marker, this protein has been used to distinguish various forms of renal cell neoplasms (Martignoni et al. 2007), and also to diagnose nephrogenic adenomas, and differentiate these from prostatic adenocarcinomas (Cossu-Rocca et al. 2009). Moreover, as mentioned above, S100A1 is downregulated in mouse, rat, and human bladder cancer (Yao et al. 2007a).

Here, we use RNA in situ hybridization and immunofluorescence analyses to characterize expression of *S100a1* mRNA and S100A1 protein in the development and homeostasis of the ureter and bladder. We find strong and specific expression of *S100a1* and S100A1 in differentiated S-cells, making them suitable markers for this cell type.

## Materials and methods

### Mice

NMRI wild-type mice were purchased in house from the animal facility of the Medizinische Hochschule Hannover. Up to four mice per cage were housed with ad libitum access to food and water under conditions of regulated temperature (22 °C) and humidity (50%) and a 12-h light/dark cycle. For timing of pregnancies, vaginal plugs were checked in the morning after mating and noon was designated as embryonic day (E) 0.5. Female mice were sacrificed by cervical dislocation. Embryos were dissected in PBS. After decapitation, trunks and dissected urogenital systems were fixed in 4% paraformaldehyde/PBS overnight and stored for several days up to 1 year in 100% methanol at − 20 °C. Specimens were subsequently transferred into paraffin wax and serial sections were cut on a microtome (Leica RM2155, Leica Biosystems, Nussloch, Germany) to a thickness of 5 µm for immunodetection and of 10 µm for RNA in situ hybridization analyses.

The mouse work was in accordance with the German Animal Welfare Legislation and approved by the local Institutional Animal Care and Research Advisory Committee and permitted by the Lower Saxony State Office for Consumer Protection and Food Safety (AZ42500/1H).

### RNA in situ hybridization analysis

Non-radioactive RNA in situ hybridization analysis of gene expression was performed on 10-μm-thick paraffin wax sections with digoxigenin-labeled antisense riboprobes as previously reported (Moorman et al. 2001). *Upk1b* and *Trp63* antisense riboprobes were obtained by transcription with the T3 RNA polymerase (#11,031,163,001, Roche, Basel, Switzerland) of linearized plasmid DNA in the presence of a Dig-RNA labeling mix (#11,277,073,910, Roche, Basel, Switzerland). For *Upk1b*, the plasmid *pFLCI.Upk1b*, which contained a 1765-bp *Upk1b* cDNA (NCBI reference sequence AK083101), was linearized with the restriction enzyme *EcoR*I (#R3101S, New England Biolabs, Ipswich, MA, USA). For *Trp63*, the plasmid *pFLCI.Trp63*, which contained a 1729-bp *Trp63* cDNA (NCBI sequence reference NM_001127263), was linearized with the restriction enzyme *Not*I (#R3189S, New England Biolabs). Probes for *Krt5* and *S100a1* were obtained from PCR products. For *Krt5*, a PCR fragment was used that was amplified from E18.5 ureter cDNA (forward primer sequence: TGTTGAACGCCGCTGACCTC, reverse primer sequence: CGCGCGTAATACGACTCACTATAGGGTGCTCAGCTTCAGCAATGGC; relating to position 8-1319 in the NCBI reference sequence NM_027011). For *S100a1*, a PCR fragment was amplified from E18.5 ureter cDNA (forward primer sequence: AGGAGGTCGGTAGGGAAAGACG, reverse primer sequence: CGCGCGTAATACGACTCACTATAGGGTGCCACTCTTGTGCCCTTGG; relating to position 14-568 in the NCBI reference sequence NM_011309). In both cases, a T7 promoter sequence was added to the 5’ end of the reverse primer in order to transcribe the PCR fragment with the T7 RNA polymerase (#M0251L, New England Biolabs, Ipswich, MA, USA) in the presence of Dig-RNA labeling mix.

For the negative control experiment, sense probes were used and no reaction was observed. Embryonic mouse inner-ear sections were used for the positive control for *S100a1*, and skin sections were used for *Krt5* and *Trp63. Upk1b* expression in the urothelium was as previously reported (data not shown).

### Immunohistochemistry and immunofluorescence

Immunofluorescence and immunohistochemical stainings were performed on paraffin wax sections of 5-µm thickness. After deparaffinization, slides were transferred in metal containers containing unmasking solution (#H3300, Vector Laboratories, Burlingame, CA, USA) in a 1:100 dilution in H_2_O. The containers were sealed and placed in boiling water for 15 min. Following this antigen retrieval step, the sections were treated for 10 min with 6% H_2_O_2_/H_2_O for blocking of endogenous peroxidases, washed three times in PBS-T (0.05% Tween-20 in PBS) and incubated for 1 h at room temperature in TNB Blocking Buffer (0.1 M TRIS–HCl pH7.5, 0.15 M NaCl and 0.5% TSA Blocking Reagent FP1012 from kit NEL702001KT, Perkin Elmer, Waltham, MA, USA). Sections were then incubated with primary antibodies in TNB buffer at room temperature for 1 h, washed three times in PBS-T, and incubated with secondary antibodies in TNB buffer at room temperature for 1 h. After washing three times in PBS-T, nuclei were stained with 4’,6-diamidino-2-phenylindole (DAPI, #6335.1, Carl Roth, Karlsruhe, Germany) and slides were mounted in IS mounting medium (#AKS-38447, Dianova, Hamburg Germany). For immunohistochemistry, the DAB substrate solution (#NEL938001EA, Perkin Elmer, Waltham, MA, USA) was used for detection after incubation with the secondary antibody.

For the negative control experiment, primary antibodies were omitted and no reaction was observed. To control the specificity of the primary antibodies, we analyzed by immunofluorescence sections of tissues previously described to express the antigens. We detected S100A1 protein in supporting cells and inner hair cells of the developing cochlea at E18.5 (Bermingham-McDonogh et al. 2006). Embryonic skin sections served as positive controls for KRT5 and ∆NP63 expression in basal cells (Croyle et al. 2011; Medawar et al. 2008). UPK1B was detected in bladder and ureter urothelium as previously reported (Gandhi et al. 2013; Bohnenpoll et al. 2017) (data not shown). Moreover, the pattern of protein expression as detected by immunofluorescence and immunohistochemistry was identical to that detected for the respective mRNAs, and thus, provided an orthogonal validation of the specificity of the four antibodies used in this study.

### Antibodies

We used the following primary antibodies for immunodetection on sections.

Keratin 5 (KRT5): Polyclonal rabbit-anti-KRT5 (clone Poly19055, #PRB-160P, Biolegend, San Diego, CA, USA) was raised against a peptide sequence derived from the C-terminus of the mouse KRT5 protein (manufacturer’s information). Basal cells of the mouse epidermis were labeled by this antibody (Croyle et al. 2011). We used the antibody in a dilution of 1:100.

∆N isoform of transformation related protein 63 (∆NP63): polyclonal rabbit-anti-∆NP63 (clone Poly6190, #619,001, Biolegend) was raised against a modified peptide and purified by antigen-affinity chromatography. Western blotting detected a single band of approximately 80 kDa in lysates of 293 cells (manufacturer’s information). Epidermal cells were immunoreactive to this antibody (Medawar et al. 2008). We used the antibody in a dilution of 1:100.

Uroplakin 1b (UPK1B): monoclonal mouse-anti-UPK1B (clone1E1, #WH0007348M2, Sigma-Aldrich, St. Louis, MO, USA) was raised against a UPK1B partial recombinant protein with GST tag corresponding to the 131-228 amino acid sequence of human UPK1B, and immunoblotting analysis of this antibody labeled a single band of 36.5 kDa (manufacturer’s information). Mouse urothelium was immunoreactive to this antibody (Bohnenpoll et al. 2017). The antibody was diluted 1:200.

S100A1: polyclonal rabbit-anti-S100A1 (#C0318-1, Acris Antibodies, Herford, Germany) was raised against a synthesized peptide derived from human S100A1. Western blot analysis of this antibody labeled a single band of approximately 10 kDa in lysates of 293 cells (manufacturer’s information). We used the antibody in a dilution of 1:200.

Fluorescent staining was performed using the following secondary antibodies in the indicated dilutions: biotinylated goat-anti-rabbit IgG (1:200; #111-065-003; Dianova, Hamburg, Germany), Alexa488-conjugated goat-anti-rabbit IgG (1:200; #A11034; Molecular Probes, Carlsbad, CA, USA), Alexa555-conjugated goat-anti-rabbit IgG (1:200; #A21428; ThermoFisher Scientific, Waltham, MA, USA) and Alexa555-conjugated goat-anti-mouse IgG (1:200; #A21422; Molecular Probes). The signal of ∆NP63 was amplified using the Tyramide Signal Amplification system (#NEL702001KT, Perkin Elmer, Waltham, MA, USA).

The following secondary antibodies were used for the immunohistochemistry: biotinylated goat-anti-rabbit IgG (1:200; #111-065-003; Dianova, Hamburg, Germany), biotin HRP-conjugated goat-anti-rabbit IgG (1:200; #111-035-045; Dianova) and biotin HRP-conjugated goat-anti-mouse IgG (1:200; #sc-2005; Santa Cruz).

### Numbers

We collected eight mice of each prenatal (E14.5, E15.5, E16.5, E17.5, E18.5) and postnatal stage (P7, P14, P40) for our expression analyses. After paraffin embedding, four specimens of each stage were used to obtain serial sections through the proximal ureter region (a row of 5-µm thickness and a row of 10 µm thickness each), the other four specimens were sectioned in a transverse manner to obtain serial bladder sections of 5- and 10-µm thickness. For each probe in the *RNA *in situ hybridization analysis and each antibody or antibody combination in the immunodetection, we used four individual sections (one each from the four different specimens) at each stage and for each tissue (ureter or bladder). To compare the expression patterns of different genes and proteins, we applied the different probes and antibodies to serial sections.

### Image documentation

Sections were photographed using either a Leica DM5000 microscope equipped with A4, L5, and TX2 filter cubes, Leica N PLAN 5x/0.12 and Leica HCX PL FLUOTAR 40x/0.75 objective lenses, and a 1.4-MP Leica DFC300FX digital camera, or a Leica DM6000 microscope equipped with A4, L5, and TX2 filter cubes, Leica N PLAN 5x/0.12 and Leica HCX PL FLUOTAR L 40x/0.60 CORR objective lenses, and a 1.4-MP Leica DFC350FX digital camera (Leica Microsystems, Wetzlar, Germany). Images were acquired with Leica Fire Cam or Leica Application Suite X (LAS X) software, and subsequently processed and composed to figures in Adobe Photoshop CS4 (Adobe, San Jose, CA, USA). All images were modified following the guidelines of the journal* Histochemistry and Cell Biology*.

## Results and discussion

Expression of *S100a1* was previously analyzed on sections of E14.5 mouse embryos as part of a large effort to map the spatial distribution of most embryonic transcripts by RNA in situ hybridization at this stage (Diez-Roux et al. 2011). The corresponding data sets have been incorporated in the Gene Expression database (GXD) at Mouse Genome Informatics (MGI) at the Jackson lab (http://www.informatics.jax.org). The assay for *S100a1* (euxassay_003348 at http://www.informatics.jax.org/assay/MGI:4827846) revealed highly specific expression in the cochlear epithelium, the ventricular myocardium, the intervertebral discs, and in the bladder epithelium. The latter site of expression prompted us to perform a detailed RNA section in situ hybridization analysis of *S100a1* expression in urothelial development and homeostasis. To get a better appreciation of the cell-type specificity of *S100a1*, we comparatively analyzed expression of *Upk1b*, *Trp63*, and *Krt5*, as these markers have routinely been used to identify S-, I-, and B-cells, respectively (Gandhi et al. 2013; Bohnenpoll et al. 2017).

**Fig. 1:**
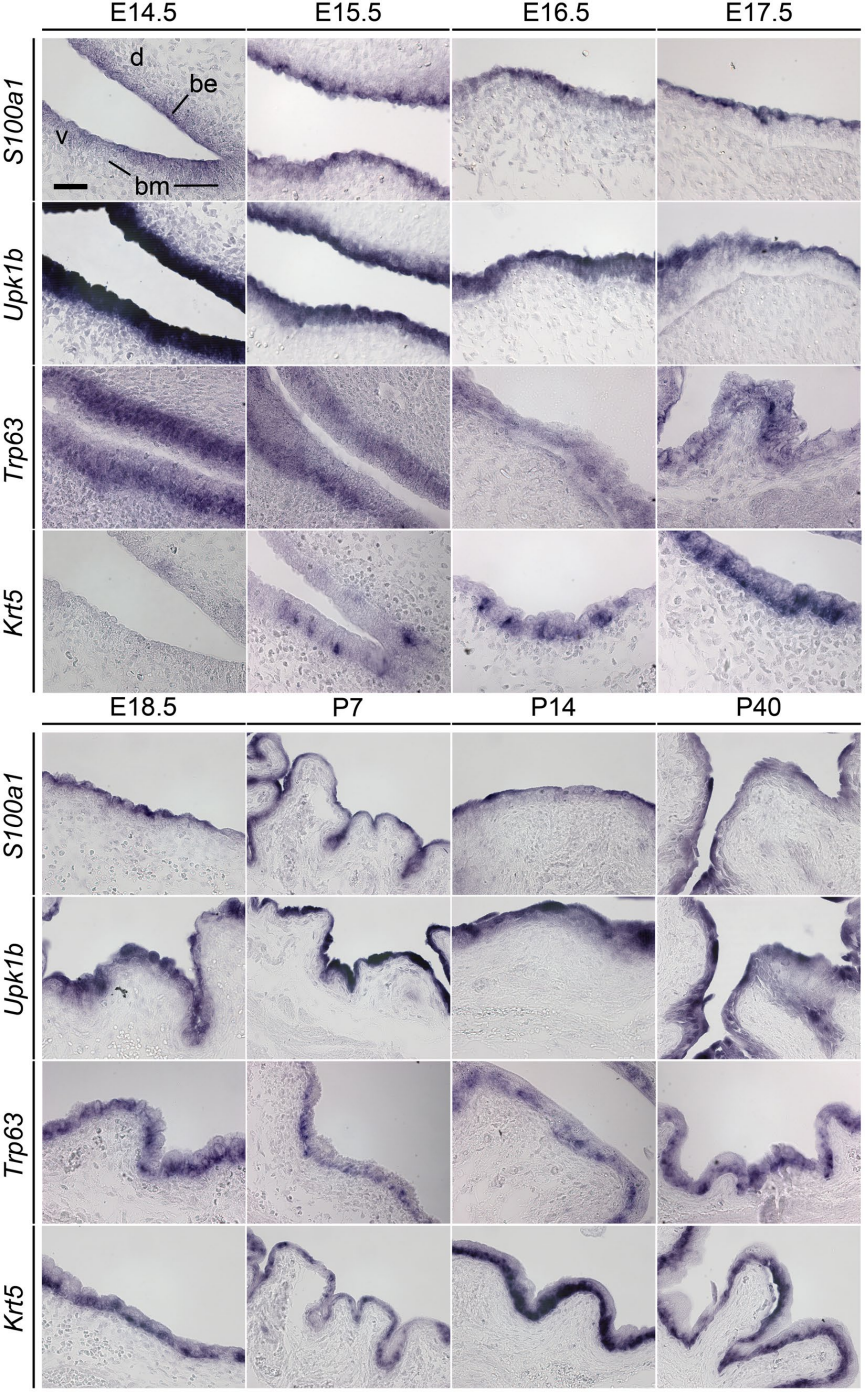
*S100a1* mRNA is restricted to luminal cells of the bladder epithelium from E14.5 of development onwards. Analysis of *S100a1* expression was performed by RNA in situ hybridization on midsagittal sections of the bladder of E14.5 to P40 mice. Expression of *Upk1b, Trp63*, and *Krt5* was comparatively analyzed to visualize layers of B-cells (*Krt5*^+^*, Trp63*^+^*, Upk1b*^−^), I-cells (*Krt5*^−^, *Trp63*^+^, *Upk1b*^*weak*+^) and S-cells (*Krt5*^−^*, Trp63*^−^*, Upk1b*^*strong*+^) in the urothelium. *be* bladder epithelium, *bm* bladder mesenchyme, *d* dorsal, *v* ventral. All images are acquired at 40 × magnification.* Scale bar* is 50 µm

In the bladder, *S100a1* expression was first detected at E14.5 by weak signals of the color reaction at the luminal site of the epithelium, which was predominantly two-layered at this stage. From E15.5 to P40, when the bladder epithelium was mostly three-layered, expression was strong and confined to the luminal cell layer. Expression of *Upk1b* was very strong in the entire bladder epithelium at E14.5. It overlapped with *S100a1* expression in the luminal layer at the following stages, but was additionally found at lower levels in cells of the intermediate layer. *Trp63* expression was confined to cells of the basal and intermediate layer from E14.5 onwards. *Krt5* expression started weakly in the bladder epithelium at E15.5. From E18.5 onwards, expression was enhanced and clearly restricted to the basal layer (Fig. 1, Fig. S1).

**Fig. 2:**
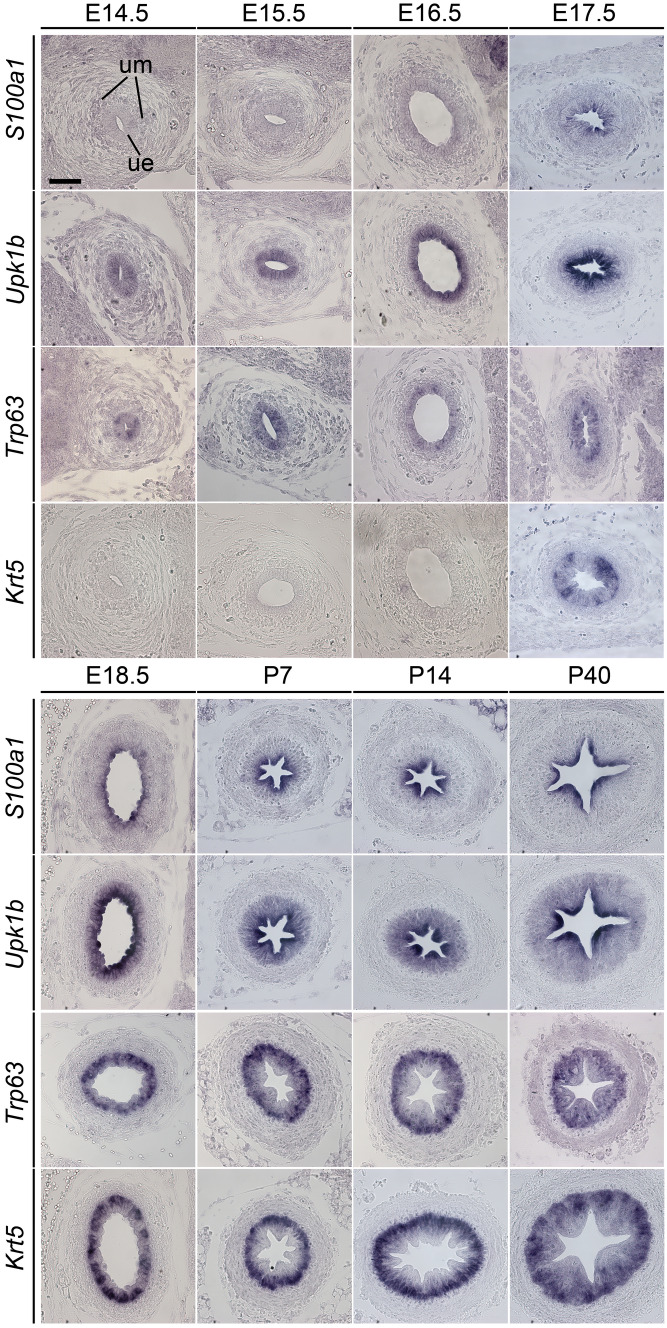
*S100a1* mRNA is restricted to luminal cells of the ureter epithelium from E17.5 of development onwards. Analysis of *S100a1* expression was performed by RNA in situ hybridization on transverse sections of the proximal ureter region of E14.5 to P40 mice. Expression of *Upk1b, Trp63*, and *Krt5* was comparatively analyzed to visualize layers of B-cells (*Krt5*^+^*, Trp63*^+^*, Upk1b*^−^), I-cells (*Krt5*^−^*, Trp63*^+^*, Upk1b*^*weak*+^) and S-cells (*Krt5*^−^*, Trp63*^−^*, Upk1b*^*strong*+^) in the urothelium. *ue* ureter epithelium, *um* ureter mesenchyme. All images are acquired at 40 × magnification.* Scale bar* is 50 µm

In ureter development, expression of *S100a1* was first detected at E17.5 in some luminal cells of the two-layered epithelium. At E18.5, when the urothelium became partly three-layered, *S100a1* expression occurred in all luminal cells and remained so until maturity (P40). At postnatal stages, *S100a1* expression outlined the arch-like shape of large luminal S-cells that resided on top of an aggregation of I- and B-cells. Expression of *Upk1b* started in the ureter epithelium at E14.5, when it was still mono-layered. From E17.5 to E18.5 onwards, expression was upregulated in the luminal cell layer while expression persisted at lower levels in the intermediate layer. *Trp63* expression commenced in the ureter epithelium at E14.5, and was found in B- and I- cells from E17.5 into adulthood. Expression of *Krt5* started in some cells of the basal layer at E17.5. From E18.5 onwards, all cells of this layer were positive for this marker (Fig. 2). Hence, *S100a1 e*xpression is confined to luminal cells in the developing and mature urothelium of the bladder and ureter. Late onset of expression, as well as exclusion from the *Trp63* and *Krt5* expression domains identifies differentiated S-cells as sites of specific *S100a1* expression.

**Fig. 3:**
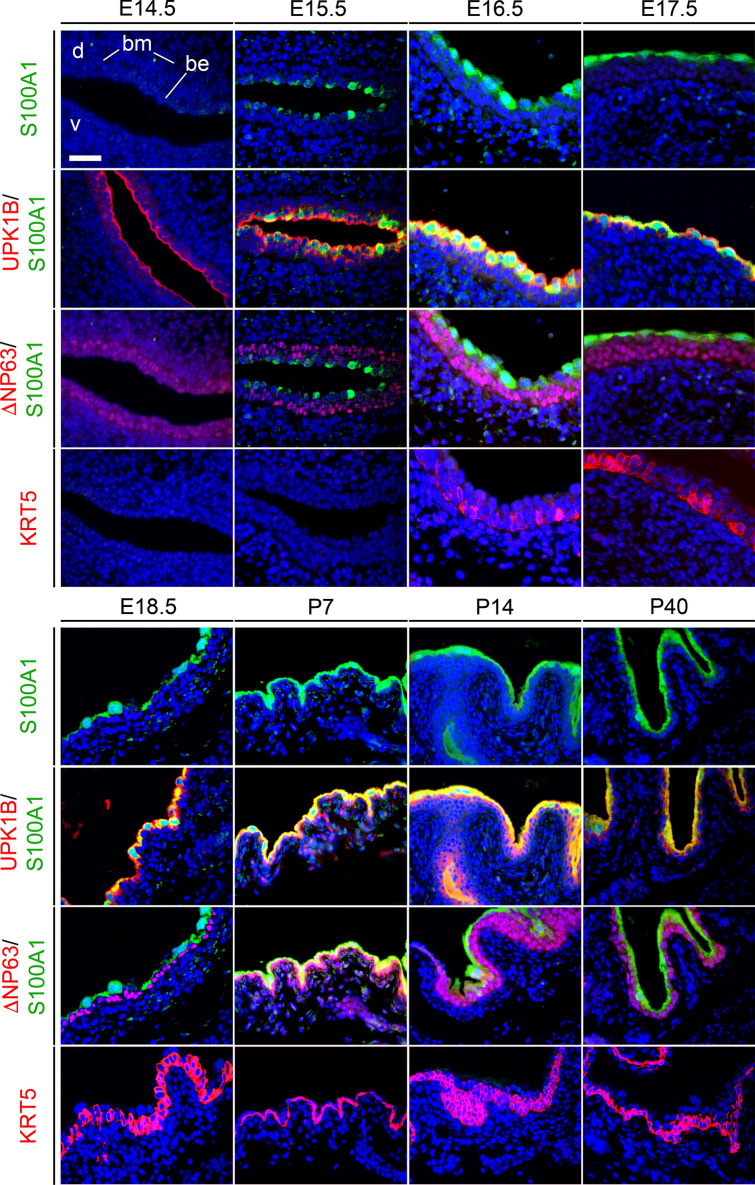
S100A1 protein is restricted to the nucleus and cytoplasm of S-cells of the bladder epithelium from E15.5 of development onwards. Analysis of S100A1 expression was performed by immunofluorescence on midsagittal sections of the bladder of E14.5 to P40 mice. Co-immunofluorescence analysis of expression of S100A1 with UPK1B and ∆NP63 and immunofluorescence analysis of KRT5 expression was performed on adjacent sections to determine the cell-type specificity. KRT5 marks the subcortical cytoplasm in B-cells, ∆NP63 marks the nuclei of B- and I-cells, while UPK1B localizes to the apical cell surface of I- and S-cells. *be* bladder epithelium, *bm* bladder mesenchyme, *d* dorsal, *v* ventral. All images are acquired at 40 × magnification.* Scale bar* is 50 µm

To determine whether S100A1 protein distribution in the urothelium follows the pattern of mRNA, we performed immunofluorescence analysis of S100A1 expression on sections of the developing and mature bladder and ureter. To more carefully determine the cell-type specificity, we additionally performed co-immunofluorescence analysis of expression of S100A1 with UPK1B and ∆NP63, and immunofluorescence analysis of KRT5 on serial sections. We used commercially available antibodies for protein detection. In the developing bladder, epithelial expression of S100A1 protein commenced in some luminal cells at E15.5, and was found in the entire luminal cell layer from E16.5 onwards. Expression was nucleocytoplasmic at all analyzed stages. Expression of S100A1 overlapped with UPK1B in luminal cells. However, UPK1B was already highly expressed at E14.5 and extended into the I-cell layer. S100A1 expression was perfectly excluded from ∆NP63-positive B- and I-cells and distinct from the KRT5-positive domain at all stages analyzed (Fig. 3, Fig. S2).

**Fig. 4:**
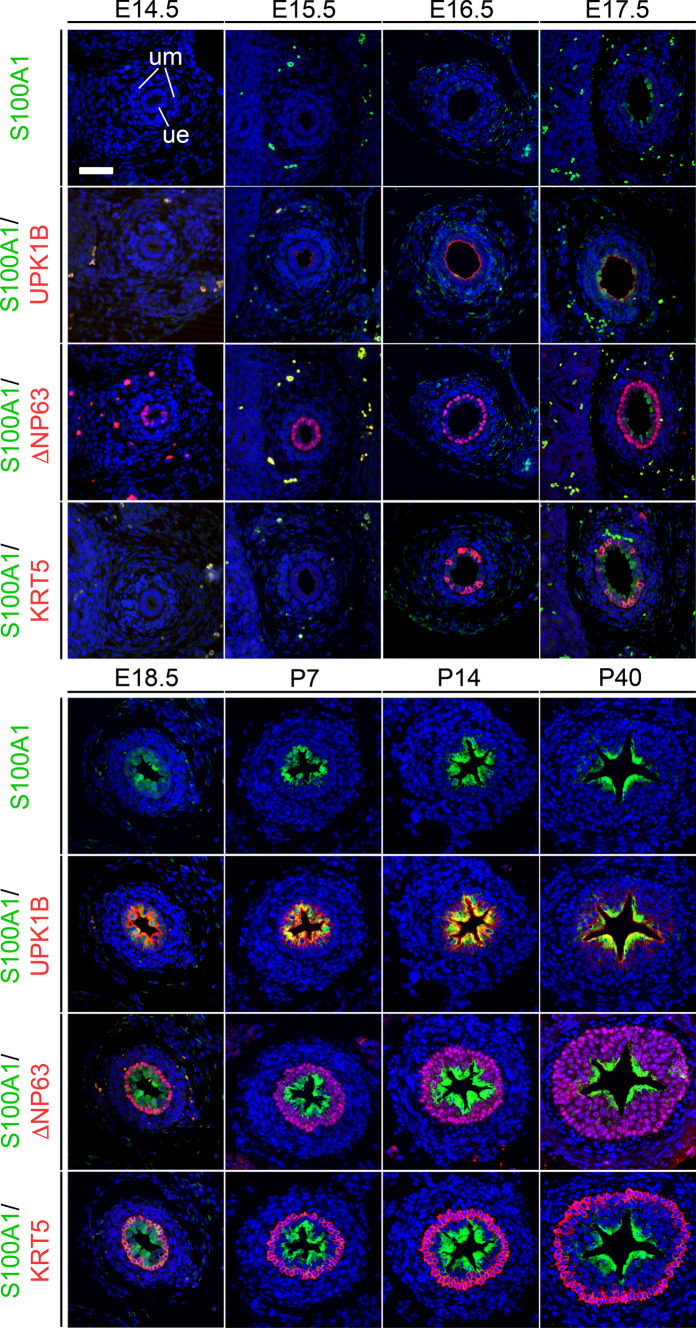
S100A1 protein is restricted to the nucleus and cytoplasm of S-cells of the ureter epithelium from E17.5 of development onwards. Analysis of S100A1 expression was performed by immunofluorescence on transverse sections of the proximal ureter of E14.5 to P40 mice. Co-immunofluorescence analysis of expression of S100A1 with UPK1B and ∆NP63 and immunofluorescence analysis of KRT5 expression was performed on adjacent sections to determine the cell-type specificity. KRT5 marks the subcortical cytoplasm in B-cells, ∆NP63 marks the nuclei of B- and I-cells, while UPK1B localizes to the cell surface of I- and S-cells. *ue* ureter epithelium, *um* ureter mesenchyme. All images are acquired at 40 × magnification.* Scale bar* is 50 µm

Expression of S100A1 in the epithelium of the ureter followed the pattern in the bladder with a delay of 2 days. At E17.5, some luminal cells showed S100A1 expression (Fig. 4). From E18.5 onwards, nucleocytoplasmic staining labeled the cell bodies of all luminal cells. Co-expression occurred with UPK1B in luminal cells while expression of ∆NP63 and KRT5 was excluded from this domain and restricted to B- and I-cells and B-cells, respectively (Fig. 4). Immunohistochemical staining confirmed these expression patterns at postnatal stages of ureter development (Fig. S3).

All of these findings show that S100A1 expression is restricted to luminal cells of the urothelium. Late onset and exclusion from ∆NP63-positive I-cells and B-cells define these cells as differentiated S-cells. S100A1 protein expression follows precisely the pattern of the *S100a1* mRNA excluding a posttranscriptional regulation, and confirming the specificity of the antibody used.

Previous work characterized Deiters’ cells and inner hair cells of the cochlea (Bermingham-McDonogh et al. 2006), ventricular cardiomyocytes (Kiewitz et al. 2000), endothelial cells (Pleger et al. 2008), and late proliferative and pre-hypertrophic chondrocytes of the growth plate (Saito et al. 2007) as sites of highly specific expression of *S100a1*/S100A1 in murine development and/or homeostasis. A possible expression in the urothelium was indicated by microarray profilings (Yao et al. 2007a, b), but was, to our knowledge, not substantiated by independent assays. Our results clearly define differentiated S-cells in the urinary drainage system as a novel site of *S100a1*/S100A1 expression, and therefore expand the range of expression domains of this calcium-binding protein.

Previous work associated loss of *S100a1* with reduced cardiac contraction rates and relaxation rate responses to beta-adrenergic stimulation (Du et al. 2002), endothelial dysfunction, and apoptosis (Teichert-Kuliszewska et al. 2015) and excitation–contraction coupling in skeletal muscle (Prosser et al. 2008). Whether S100A1 is functionally involved in the terminal differentiation of S-cells and/or the maintenance of this highly specific and relevant cell type, e.g., by controlling the energy metabolism or biosynthetic activity is a possibility that may be addressed by future research.

Nucleocytoplasmic localization of the protein allows the unambiguous identification of the large and extended shape of S-cells particularly in the ureter where these cells cover a grape-like accumulation of I- and B-cells. S100A1 as a marker for urothelial S-cells in the mouse is superior to KRT20 which has only been described in mature S-cells in the bladder (Erman et al. 2001), and to UPKs which are predominantly localized on the cell surface, are expressed at low levels in I-cells, and are activated prior to terminal differentiation of S-cells.

S100A1 was suggested as a useful marker for the detection of ovarian cancer (Hibbs et al. 2004; Tian et al. 2017) the distinction of nephrogenic adenoma from prostatic adenocarcinoma (Cossu-Rocca et al. 2009), the prognosis of relapse-free survival in patients suffering from endometrial cancer (DeRycke et al. 2009), and the diagnosis and prognosis of papillary thyroid carcinoma (Wang et al. 2021). It will be interesting to explore whether S100A1 presents a suitable marker for recognizing urothelial carcinomas and distinguishing their subtypes in the murine and the human context.

## Supplementary Information

Below is the link to the electronic supplementary material.

Supplementary file1 (PDF 3661 KB)

## Data Availability

All data generated or analyzed during this study are included in this published article (and its supplementary information files). Additional information is available from the corresponding author upon reasonable request.
