# Supplementary material for: S100A1 expression characterizes terminally differentiated superficial cells in the urothelium of the murine bladder and ureter
Source: Histochem Cell Biol. 2022 Jun 1;158(4):389–99. doi: 10.1007/s00418-022-02120-1 (PMC9512885; doi:10.1007/s00418-022-02120-1)
Supplement: Supplementary file 1 — Supplementary file1 (PDF 3661 KB) [file 418_2022_2120_MOESM1_ESM.pdf]

Supplementary Figures

for

S100A1 expression characterizes terminally differentiated superficial cells in the urothelium of the murine bladder and ureter

Fairouz Qasrawi[1], Max Meuser[1], Finja Lehnhoff[1], Marjenna Schulte[1] and Andreas Kispert[1,§]

[1]Institut für Molekularbiologie, OE5250, Medizinische Hochschule Hannover, Carl-Neuberg-Str. 1, D-30625 Hannover, Germany.

Fairouz Qasrawi, Max Meuser and Finja Lehnhoff contributed equally

§ Address correspondence to: Andreas Kispert, E-Mail: kispert.andreas@mh-hannover.de, ORCID-ID: 0000-0002-8154-0257

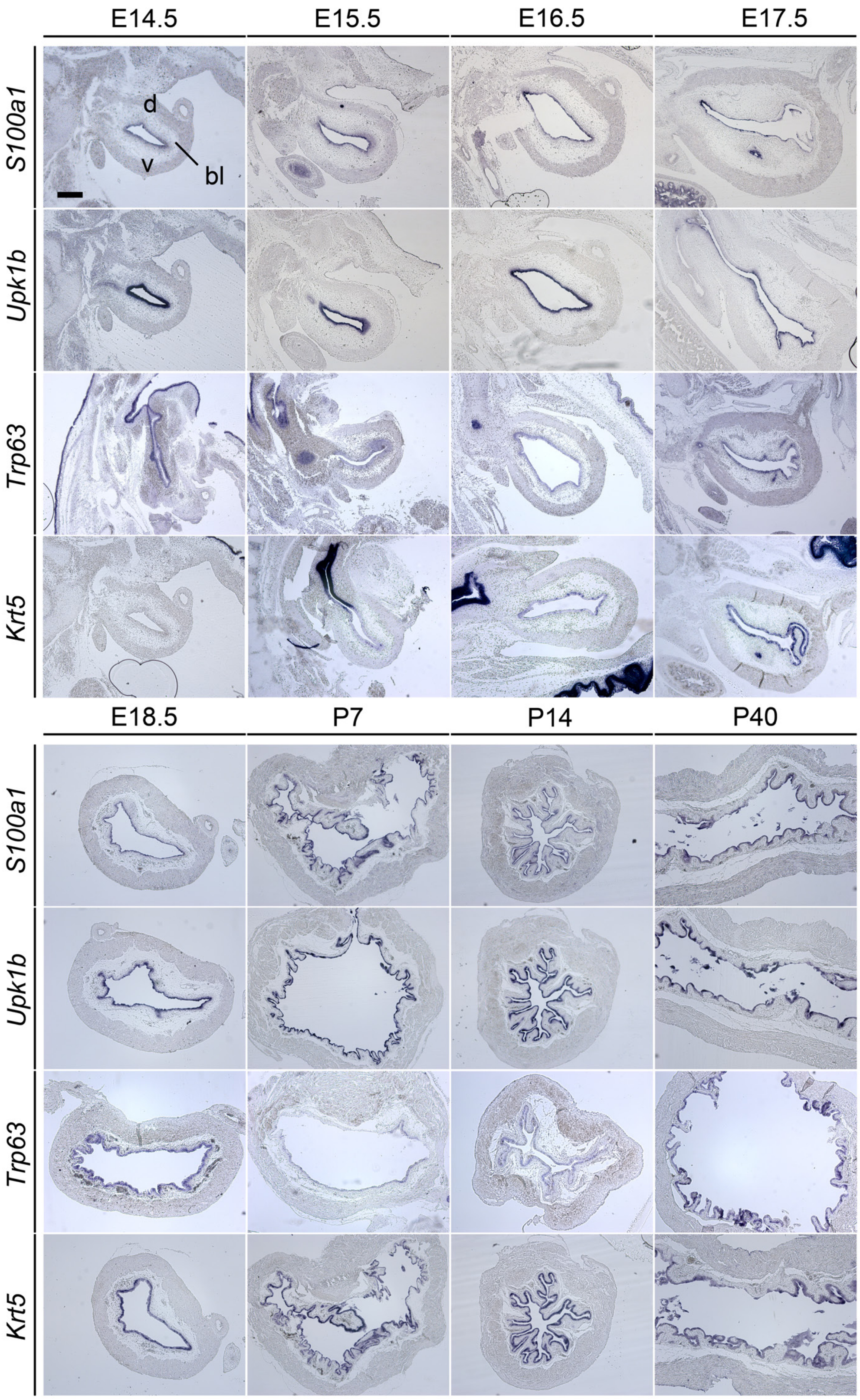

Fig. S1 *S100a1* mRNA is restricted to luminal cells of the bladder epithelium from E14.5 of development onwards. Analysis of *S100a1* expression was performed by RNA *in situ* hybridization on midsagittal sections of the bladder of E14.5 to P40 mice. Expression of *Upk1b, Trp63* and *Krt5* was comparatively analyzed to visualize layers of B-cells (*Krt5*$^{+}$,*Trp63*$^{+}$, *Upk1b*$^{-}$), I-cells (*Krt5*$^{-}$, *Trp63*$^{+}$, *Upk1b*$^{weak+}$) and S-cells (*Krt5*$^{-}$, *Trp63*$^{-}$, *Upk1b*$^{strong+}$) in the urothelium. bl, bladder; d dorsal; v, ventral. All images are acquired at 5× magnification. Scale bar is 300 μm

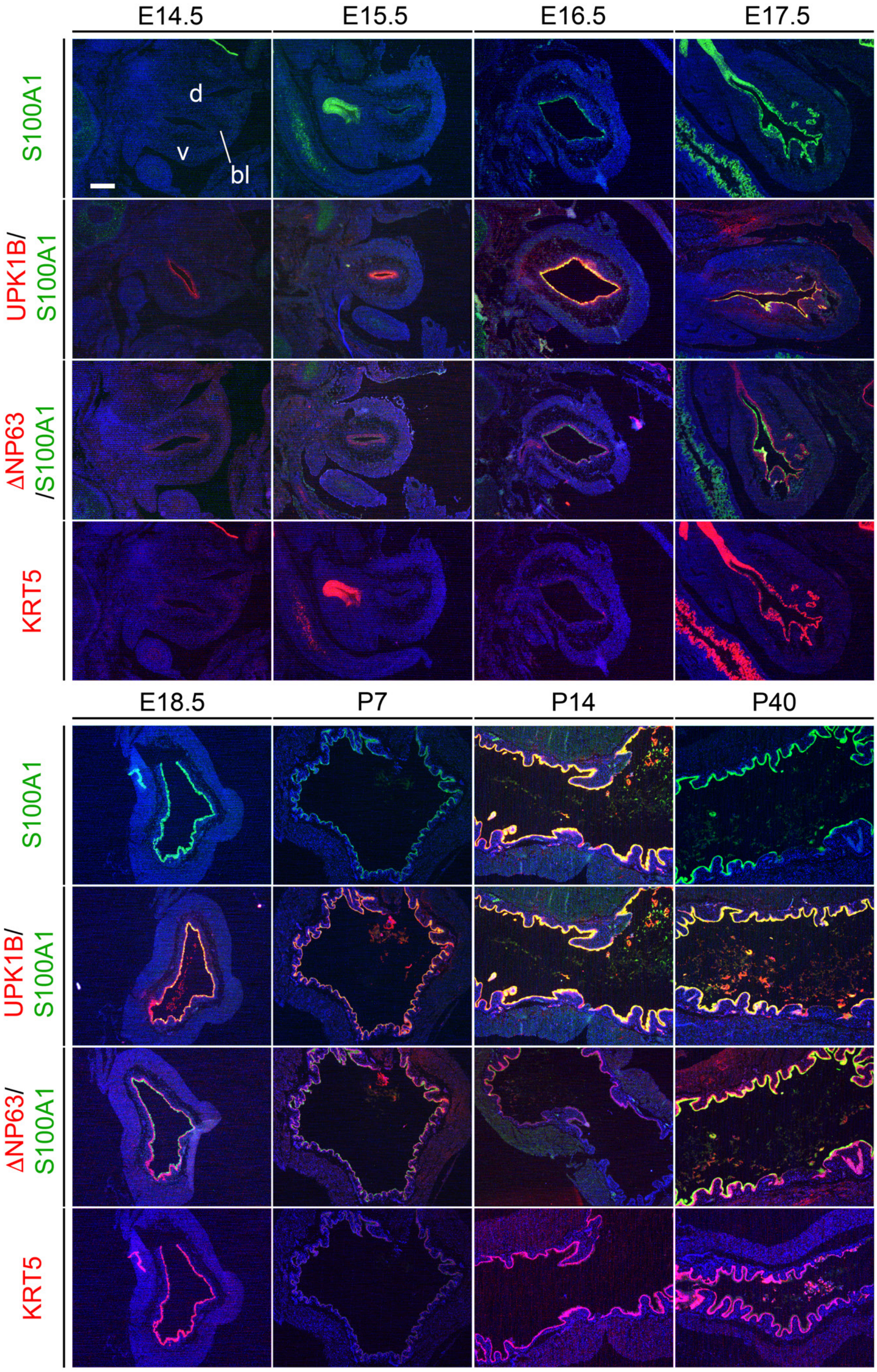

Fig. S2 S100A1 protein is restricted to luminal cells of the bladder epithelium from E15.5 of development onwards. Analysis of S100A1 expression was performed by immunofluorescence on midsagital sections of the bladder of E14.5 to P40 mice. Co-immunofluorescence analysis of expression of S100A1 with UPK1B and ΔNP63 and immunofluorescence analysis of KRT5 expression was performed on adjacent sections to determine the cell-type specificity. KRT5 marks the subcortical cytoplasm in B-cells, ΔNP63 marks the nuclei of B- and I-cells, while UPK1B localizes to the apical cell surface of I- and S-cells. be, bladder epithelium; bm, bladder mesenchyme; d dorsal; v, ventral. All images are acquired at 5× magnification. Scale bar is 300 μm

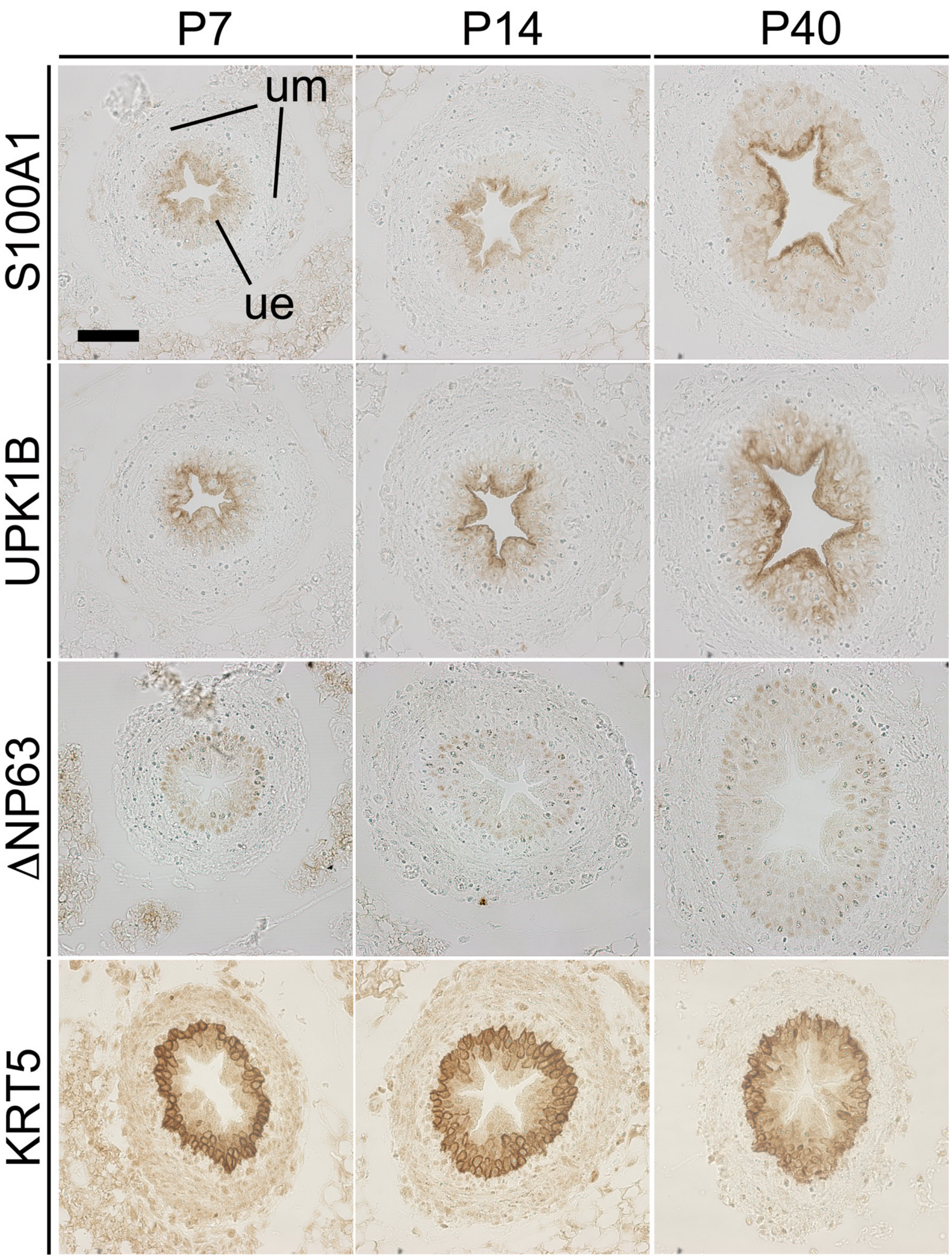

Fig. S3 S100A1 protein labels luminal cells of the ureter epithelium at postnatal stages. Analysis of S100A1 expression was performed by immunohistochemistry on transverse sections of the proximal ureter of P7, P14 and P40 mice. Expression of UPK1B, ΔNP63 and KRT5 was comparatively analyzed. ue, ureter epithelium; um, ureter mesenchyme. All images are acquired at 40× magnification. Scale bar is 50 μm
